# Circulation of influenza and other respiratory viruses during the COVID-19 pandemic in Australia and New Zealand, 2020–2021

**DOI:** 10.5365/wpsar.2023,14.3.948

**Published:** 2023-07-27

**Authors:** Genevieve K O’Neill, Janette Taylor, Jen Kok, Dominic E Dwyer, Meik Dilcher, Harry Hua, Avram Levy, David Smith, Cara A Minney-Smith, Timothy Wood, Lauren Jelley, Q Sue Huang, Adrian Trenholme, Gary McAuliffe, Ian Barr, Sheena G Sullivan

**Affiliations:** a WHO Collaborating Centre for Reference and Research on Influenza, Royal Melbourne Hospital, Melbourne, Victoria, Australia.; b Centre for Infectious Diseases and Microbiology Laboratory Services, New South Wales Health Pathology-Institute of Clinical Pathology and Medical Research, Westmead Hospital, Westmead, New South Wales, Australia.; c Canterbury Health Laboratories, Christchurch, New Zealand.; d PathWest Laboratory Medicine WA, Nedlands, Western Australia, Australia.; e Infection and Immunity, School of Biomedical Sciences, University of Western Australia, Perth, Western Australia, Australia.; f Faculty of Health and Medical Sciences, University of Western Australia, Nedlands, Western Australia, Australia.; g Institute of Environmental Science and Research, Wellington, New Zealand.; h Counties Manukau District Health Board, Auckland, New Zealand.; i Virology and Immunology Department, LabPLUS, Auckland City Hospital, Auckland District Health Board, Auckland, New Zealand.; j Department of Microbiology and Immunology, University of Melbourne, Peter Doherty Institute for Infection and Immunity, Melbourne, Victoria, Australia.; k Department of Infectious Diseases and Centre for Epidemiology and Biostatistics, University of Melbourne, Melbourne, Victoria, Australia.

## Abstract

**Objective:**

Circulation patterns of influenza and other respiratory viruses have been globally disrupted since the emergence of coronavirus disease (COVID-19) and the introduction of public health and social measures (PHSMs) aimed at reducing severe acute respiratory syndrome coronavirus 2 (SARS-CoV-2) transmission.

**Methods:**

We reviewed respiratory virus laboratory data, Google mobility data and PHSMs in five geographically diverse regions in Australia and New Zealand. We also described respiratory virus activity from January 2017 to August 2021.

**Results:**

We observed a change in the prevalence of circulating respiratory viruses following the emergence of SARS-CoV-2 in early 2020. Influenza activity levels were very low in all regions, lower than those recorded in 2017–2019, with less than 1% of laboratory samples testing positive for influenza virus. In contrast, rates of human rhinovirus infection were increased. Respiratory syncytial virus (RSV) activity was delayed; however, once it returned, most regions experienced activity levels well above those seen in 2017–2019. The timing of the resurgence in the circulation of both rhinovirus and RSV differed within and between the two countries.

**Discussion:**

The findings of this study suggest that as domestic and international borders are opened up and other COVID-19 PHSMs are lifted, clinicians and public health professionals should be prepared for resurgences in influenza and other respiratory viruses. Recent patterns in RSV activity suggest that these resurgences in non-COVID-19 viruses have the potential to occur out of season and with increased impact.

The Australian and New Zealand governments’ response to the coronavirus disease (COVID-19) pandemic has been described as “hard and fast,” with the initial aim of elimination. (*1*) Both countries swiftly introduced a range of public health and social measures (PHSMs), such as physical distancing, mask use, school closures, border closures and travel restrictions. The stringency of these PHSMs fluctuated in both countries throughout 2020–2021, in response to local COVID-19 outbreaks. During this time, circulating patterns of influenza and other respiratory viruses changed dramatically. (*2*)

Respiratory viruses cause significant morbidity and mortality. (*3*, *4*) Influenza is known to cause severe illness in elderly adults, (*3*) while human parainfluenza virus types 1–3 (PIV-1–3) have the potential to cause severe disease in infants and children. (*5*) However, human PIV type 4 usually only causes mild or asymptomatic infections. Respiratory syncytial virus (RSV) and human metapneumovirus (hMPV) can cause severe disease in infants, children, elderly adults and immunocompromised patients. (*6*) Human rhinoviruses, one of the most commonly reported viruses in childcare centres where it is not uncommon for children to experience multiple infections in the same season, are almost invariably associated with mild disease. (*7*) Likewise, human adenoviruses usually only cause mild symptoms, but they have occasionally been associated with severe nosocomial outbreaks. (*8*)

In the temperate zones, most of these common respiratory viruses have tended to exhibit predictable seasonal patterns, with activity levels peaking in the winter months. Respiratory virus surveillance systems are designed to correspond to this seasonality and may be activated only during the winter months; however, most systems retain the capacity to be reactivated out of season to detect and monitor unexpected outbreaks. (*2*) The emergence of severe acute respiratory syndrome coronavirus 2 (SARS-CoV-2), currently characterized as an endemic respiratory virus without a clearly defined seasonality, has highlighted the importance of having systems capable of detecting and monitoring any variations in seasonal respiratory virus activity to inform clinical management, public health planning and resource allocation. Here we describe respiratory virus activity from January 2020 to August 2021 in selected regions of Australia and New Zealand.

## Methods

### Study populations

**Fig. 1:**
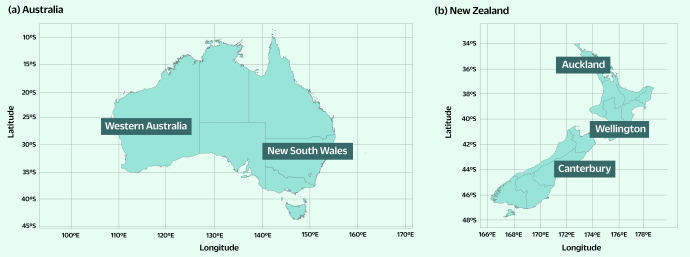
Geographical representation of the five study regions in (a) Australia and (b) New Zealand

The study was conducted using data from five regions: New South Wales (NSW) and Western Australia (WA) in Australia; and Auckland, Canterbury and Wellington in New Zealand. These represent geographically diverse locations (**Fig. 1**) that experienced different levels of COVID-19 restrictions and SARS-CoV-2 activity.

[**insert** Figure 1]

### Laboratory-based surveillance

Laboratory data routinely collected as part of regional public health surveillance were prospectively collated. Data were provided by (i) Centre for Infectious Diseases and Microbiology Laboratory Services, NSW Health Pathology-Institute of Clinical Pathology and Microbiology Research (ICPMR), Westmead, NSW; (ii) PathWest Laboratory Medicine, WA; (iii) Institute of Environmental Science and Research (ESR), Wellington, and Auckland and Counties Manukau District Health Boards, Auckland; and (iv) Canterbury Health Laboratories, Christchurch. Respiratory specimens underwent nucleic acid amplification testing (NAAT) using semiquantitative real-time reverse transcription polymerase chain reaction (PCR) or transcription-mediated amplification testing. In addition, respiratory specimens underwent rapid PCR testing at Canterbury Health Laboratories for influenza and RSV, and rapid PCR testing for influenza at ESR.

ICPMR and PathWest laboratories are major diagnostic hubs that provide state-wide testing for respiratory viruses, in both hospitalized and community populations. ESR data included laboratory-based respiratory virus testing results from specimens ordered by clinicians for hospital inpatients and outpatients during normal clinical practice from two hospital laboratories in Auckland (LabPLUS and Counties Manukau District Health Board Laboratory). Data from ESR also included testing results (severe acute respiratory infection, influenza-like illness [ILI]) from its National Influenza Centre for public health surveillance and from Wellington-based community cohorts. Canterbury Health Laboratories is a reference laboratory that provides services to general practice surgeries and hospitals in the Canterbury District Health Board region. The proportion of positive tests (referred to as virus activity) was calculated and smoothed using a 3-week, centred moving average.

### COVID-19 notification data

Australian and New Zealand COVID-19 notification data were sourced from Our World in Data (https://ourworldindata.org) on 7 March 2022. (*9*)

### Public health and social measures

Different PHSMs were adopted by the five study regions (NSW, WA, Auckland, Canterbury and Wellington) (**Supplementary Table 1**). Moreover, the intensity of these measures changed throughout the study period. (*10*, *11*) Google mobility data sourced on 6 October 2021 were used as an indicator of compliance with PHSMs (*12*) Using mapping apps, Google mobility data capture the daily movements of people with an Android device relative to a baseline period. Google provides these data in the form of COVID-19 community mobility reports, expressed as a daily percentage change relative to a 5-week baseline period (3 January–6 February 2020) for six key mobility categories. (*12*) For each of the five regions, the daily average percentage change for three mobility categories (retail and recreation, transit stations, workplaces) was calculated. The other mobility categories (i.e. grocery and pharmacy, parks, residential) were excluded, as these activities were allowed even during periods of the most restrictive PHSMs in all regions and thus unlikely to reflect PHSM compliance. The proportion of change from baseline was smoothed using a 3-week, centred moving average, and plotted in a time series along with local PHSMs and respiratory virus activity.

Data were analysed using R version 4.0.4 (R Project for Statistical Computing).

## Results

**Fig. 2:**
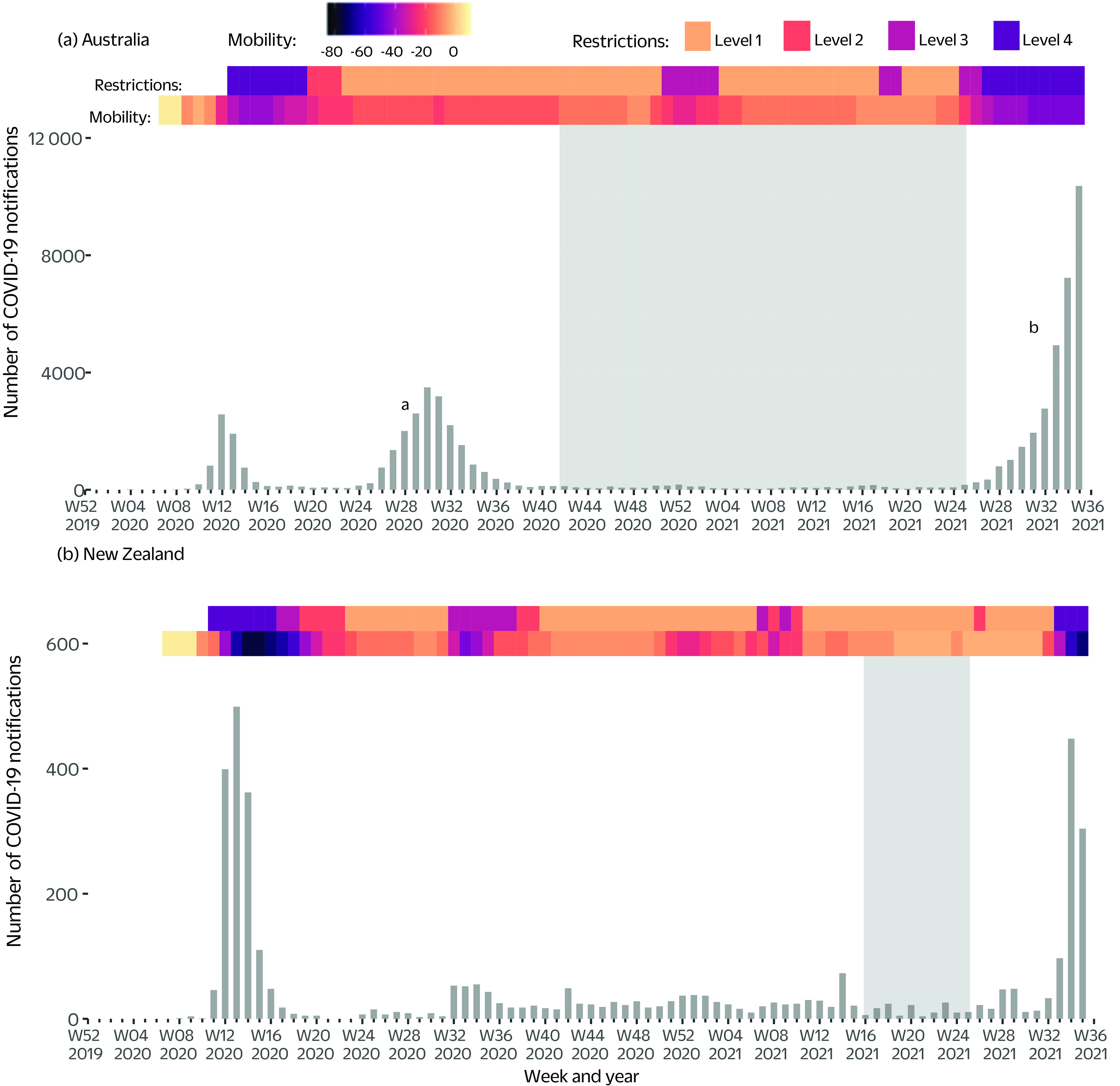
New weekly COVID-19 notifications in (a) Australia and (b) New Zealand against mobility data and level of restrictions, 2020–2021 (up to week 36, 2021)

**Fig. 3:**
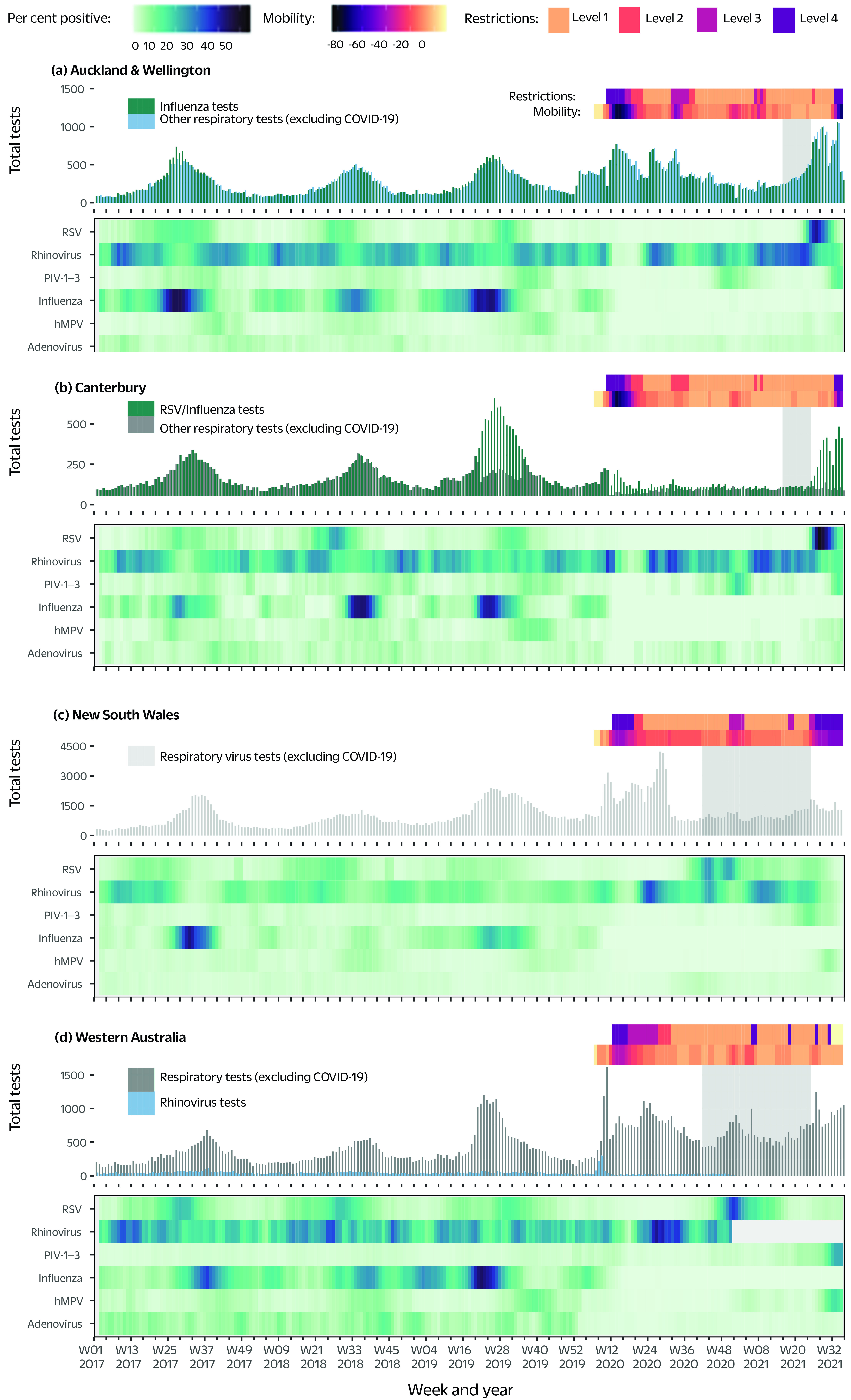
Seasonal respiratory virus activity in selected regions of (a, b) New Zealand and (c, d) Australia against mobility data and level of restrictions, 2017–2021 (up to week 36, 2021)

The amount of respiratory specimen testing for influenza and other respiratory viruses varied by region and year. In February 2020, both countries experienced their first wave of COVID-19 notifications (**Fig. 2**), and in response, respiratory virus testing (excluding COVID-19) in all regions increased (**Fig. 3**). By August 2021, the end of our study period, respiratory virus testing rates in all regions had not returned to their usual seasonal patterns.

[**insert** Figure 2]

[**insert** Figure 3]

In all regions, influenza was the most commonly detected respiratory virus in 2017–2019, followed by rhinoviruses and RSV. In contrast, between April 2020 and August 2021, the most commonly detected (non-COVID-19) respiratory viruses were rhinoviruses and RSV, followed by PIV-1–3 in all regions. All of these non-COVID-19 respiratory virus outbreaks occurred in the absence of any substantial SARS-CoV-2 circulation (**Fig. 2**).

Mobility data showed that mobility in all five regions dropped below baseline levels before or shortly after the introduction of COVID-19 restrictions. In NSW, restrictions were introduced on 18 March 2020 (week 11), with a reduction in mobility observed in week 11 (**Fig. 3**). In WA, restrictions were introduced on 23 March 2020 (week 12), with a reduction in mobility observed from week 10. In Auckland, Wellington and Canterbury, restrictions were introduced on 21 March 2020 (week 11), with a reduction in mobility observed in week 12 (**Fig. 3**, **Supplementary Table 1**). (*11*) The only region reporting mobility above baseline levels after the introduction of restrictions was Canterbury, which peaked at 2% above baseline in week 19, 2021 (**Fig. 3**).

Overall, mobility data showed an inverse relationship with restriction levels, with mobility decreasing as restrictions increased in all regions (**Fig. 3**). In all regions, outbreaks of rhinoviruses, RSV, PIV-1–3 and adenoviruses in 2020–2021 coincided with lower levels of restrictions and higher levels of population mobility (**Fig. 3**). In contrast, increases in hMPV activity were for the most part observed at a time when mobility levels were decreasing; the only exception was WA, where hMPV activity increased as mobility increased (**Fig. 3**).

## Discussion

We observed variable respiratory virus activity in five regions of Australia and New Zealand following the start of the COVID-19 pandemic in February 2020, with the notable exception of influenza, which did not circulate in either Australia or New Zealand during the pandemic period studied. After the relaxation of PHSMs in May 2020, adenovirus and rhinovirus activity increased above 2017–2019 levels in most regions (**Fig. 3**). In comparison, hMPV activity only began to rise in the autumn/winter of 2021 (i.e. May to August) after 18 months of low activity. RSV and PIV-1–3 both showed a delay in their usual seasonal pattern. Once they returned, both viruses experienced rates of activity above their 2017–2019 levels; however, in both cases, the timing of the increased activity differed between the two countries. Below we discuss some of the factors that likely contributed to these observed patterns in post-pandemic non-COVID-19 respiratory virus activity.

The nature of the relationship between infectious disease activity and mobility – specifically how, where and when people move – is well established. (*13*) Generally speaking, as mobility increases, the number of contacts or interactions between contagious and susceptible individuals also increases, resulting in an increase in virus prevalence and an outbreak of a communicable disease. The reverse is also generally true; as mobility declines, so too does respiratory virus activity. We observed reductions in population mobility coinciding with increased PHSMs in all five regions since March 2020. Overall, mobility remained below the pre-pandemic baseline in all regions, apart from a short period in Canterbury.

An early measure to contain the spread of SARS-CoV-2 was the closure of schools, with classes moving to online and home learning. Schools in New Zealand and WA reopened on 18 May 2020, while in NSW schools never officially closed but students were encouraged to learn from home from 24 March to 22 May 2020. (*10*, *11*) The initial decline and subsequent resurgence of adenoviruses and rhinoviruses in 2020 corresponded with the relaxation of some PHSMs and the reopening of schools, supporting the role of children in their circulation. Interestingly, the subsequent reintroduction of more restrictive PHSMs including school closures did not appear to reduce their activity. Possible explanations for this observation include the non-enveloped features of rhinoviruses and adenoviruses (which may make them more durable), (*14*) and post-COVID-19 changes in respiratory testing patterns.

Non-enveloped viruses such as adenoviruses and rhinoviruses are unique among the viruses included in this study in that they have some heat-resistant properties and can survive some disinfection processes including handwashing. (*14*) In addition, it has been suggested that surgical face masks are not particularly effective at reducing the emission of rhinovirus particles (aerosols and droplets). (*15*) Given that in both countries mandatory mask use was limited to times with stricter restrictions and only recommended at other times (**Supplementary Table 1**), it seems likely that at least some of the COVID-19 infection control measures may have been less effective against adenovirus and rhinovirus transmission and this allowed these viruses to continue to circulate despite the reintroduction of more restrictive PHSMs

Changes in the volume of, and in the way in which the public accessed, respiratory virus testing may also have played a role in the observed patterns of adenovirus and rhinovirus activity. (*16*) In both countries, COVID-19 testing centres were established within and outside medical facilities, providing the community with free and rapid access to SARS-CoV-2 testing. While most of the new capacity provided by the private laboratories was restricted to SARS-CoV-2 only, several public health laboratories continued to screen samples for other respiratory viruses. (*17*, *18*)

Although all regions experienced a reduction in testing capacity during the early stage of the pandemic due to pressures on staff availability, testing reagents and equipment, (*17*, *18*) NSW, WA, Auckland and Wellington were able to increase their testing rates for other respiratory viruses. The introduction of rapid SARS-CoV-2 NAAT and multiplex NAAT, which target SARS-CoV-2 and other respiratory viruses simultaneously, likely played a role in enabling these regions to not only maintain but even increase their testing rates for non-SARS-CoV-2 respiratory viruses. (*19*) The relatively lower levels of testing in Canterbury can be attributed to the inclusion of data captured by public health surveillance programmes in the Auckland and Wellington data sets, whereas the Canterbury data set only included the results of testing in hospitals and general practice surgeries. Additionally, after the closure of international borders in New Zealand, Canterbury implemented a stricter respiratory virus testing triaging system, which limited availability of multiplex respiratory virus testing to hospitalized patients. However, while increased testing may explain the increased detection of rhinoviruses and adenoviruses in 2020, it is unlikely to be a major contributor to the observed resurgence in adenovirus and rhinovirus activity, given the concurrent increase in activity of both viruses in Canterbury, where respiratory virus testing rates were significantly lower than in Auckland and Wellington.

We consider it unlikely that stringent PHSMs alone resulted in substantial decreases in the transmission of influenza and some other respiratory viruses that we observed during 2020 and suggest that viral displacement and interference could have played a contributory role. Viral interference between respiratory viruses, whereby two viruses interact within a host, has been well documented. (*20*, *21*) For example, a rhinovirus outbreak in 2009 is believed to have delayed the arrival of the 2009 influenza A (H1N1) pandemic in some European countries. (*22*, *23*) Evidence of similar interactions between SARS-CoV-2 and other viruses is now beginning to emerge; one recent study found prior infection with rhinoviruses reduced the ability of SARS-CoV-2 to replicate in respiratory tract epithelium, (*24*) suggesting that the immune-mediated effects of rhinoviruses, a common and generally mild respiratory virus, might provide a low level of protection against both SARS-CoV-2 infection and severe COVID-19 disease. Similarly, viral displacement and interference may have contributed to the delayed rise in hMPV, a rise that we observed only towards the end of the study period at a time when RSV activity was either low (as in NSW and WA) or waning after an outbreak (as in Auckland, Canterbury and Wellington). Evidence to support this hypothesis comes from a pre-pandemic study on circulating respiratory viruses conducted in Victoria, Australia, which found that RSV protected against a subsequent hMPV infection. (*21*)

Domestic and international travel have previously been linked to the introduction and subsequent spread of influenza, (*25*, *26*) and our data suggest that the lifting of restrictions may have played a role in the spread of several non-COVID-19 respiratory viruses. After March 2020, international travel was severely limited in both countries, and strict border restrictions were in place throughout 2020–2021 (**Supplementary Table 1**). Apart from short-lived travel bubbles between some Australian states and New Zealand, both countries required all international arrivals to undergo government-managed 14-day quarantine. Additionally, during COVID-19 outbreaks, domestic travel was severely restricted, with travellers from locations with current COVID-19 outbreaks prevented from entering another region or required to self-isolate on arrival for 14 days and to restrict movement within these cities (**Supplementary Table 1**). However, shortly after New Zealand allowed people from Australia to enter the country without quarantining, a resurgence in RSV activity was observed, leading to speculation that Australian travellers reseeded RSV in New Zealand in April 2021.

Before the travel bubble with Australia was introduced, RSV activity was below 1% in New Zealand but above 5% in NSW and WA. This, coupled with the fact that there is no known non-human reservoir for RSV, (*27*) does suggest international travel was the most likely cause of the increased RSV activity observed in New Zealand in 2021. Sequencing data support the hypothesis that RSV was imported from NSW into Victoria, Australia in 2020 after the second COVID-19 wave. (*28*) However, the lifting of travel restrictions does not explain the rise of RSV in WA in 2020 or the second out-of-season peak that was seen at the end of 2021. (*29*) In contrast, RSV activity in NSW returned to its normal seasonality in 2021, with no out-of-season activity reported during the 2021–2022 summer season. (*30*)

In both countries, high influenza vaccination rates could have contributed to the observed low influenza activity. This is unlikely to have been a key factor, as in 2020, Australia and New Zealand reported their highest-ever rates of influenza vaccinations. (*31*, *32*) However, influenza vaccination rates were significantly lower in both countries in 2021, (*31*, *32*) possibly due to interference with COVID-19 vaccination campaigns and complacency due to low influenza activity. It is highly improbable that circulating influenza viruses were less transmissible as influenza viruses have continued to circulate in various parts of the world such as Western Africa and China since the start of the COVID-19 outbreak. (*33*)

Past pandemics have shown that we need to be prepared for unpredictable resurgences in respiratory viruses, including out-of-season outbreaks. (*22*, *34*) In this respect at least, the COVID-19 pandemic is not exceptional. The observed out-of-season outbreaks of RSV (*28*) were not entirely unexpected given that modelling studies had predicted an RSV resurgence after the relaxation of PHSMs It is also likely that RSV seasonality will take several years to return to its pre-pandemic pattern. (*35*) As fewer people are exposed to (and infected with) respiratory viruses, population immunity decreases and the chance of more substantial outbreaks increases. When influenza returns, low rates of influenza vaccination and a possible mismatch between circulating viruses and vaccines (due to low influenza numbers in 2020 and 2021 and geographic differences in virus circulation) could increase population susceptibility and lead to larger outbreaks with more cases of severe disease. As COVID-19 vaccination rates rise and countries relax PHSMs, including easing of travel restrictions and reopening borders, respiratory virus activity will need to be closely monitored. (*36*, *37*)

This study has several limitations. First, laboratory surveillance is passive and subject to selection bias due to the subjectivity of health-care providers testing patients and variations in health-seeking behaviours. Moreover, rapid PCR testing at Canterbury Health Laboratories and reported by ESR only detects influenza A/B viruses and RSV, whereas standard multiplex PCR testing used by the other surveillance programmes and laboratories is capable of detecting a wider range of clinically relevant respiratory viruses, including human rhinoviruses, hMPV, PIV and human adenoviruses. Additionally, it is difficult to determine the proportion of testing that was provided by public health laboratories as testing conducted by private pathology services was not included in this analysis. The impact of changes in testing processes and reporting mechanisms at public health laboratories overburdened with SARS-CoV-2 testing over the period of our analysis is also difficult to assess.

Finally, it is unlikely that our baseline mobility is a true reflection of pre-pandemic mobility. Google mobility data reflected the movement of people with an Android device using mapping apps relative to a 5-week baseline period (3 January–6 February 2020). Google mobility baseline data in Australia were collected during the 2019–2020 bushfire season, with active bushfires in NSW and WA occurring during the entire 5-week baseline period. Furthermore, the baseline period largely coincided with school holidays in Australia and New Zealand, a period during which mobility patterns are different. (*38*)

## Conclusion

Seasonal respiratory virus circulation patterns have been disrupted during the COVID-19 pandemic. Epidemics of some viruses such as RSV have been observed out of season and with greater intensity than in the past. It is likely to take several years for respiratory viruses to return to their characteristic, pre-pandemic seasonal patterns. During this period, health-care systems should not rely on historical seasonal patterns to inform resource allocation and interventions. This study also serves to underscore the importance of surveillance systems with real-time data that can signal relevant epidemiological information back to clinicians and so prompt timely public health action and response.
